# Integrative Analysis of lncRNAs, miRNAs, and mRNA-Associated ceRNA Network in an Atopic Dermatitis Recurrence Model

**DOI:** 10.3390/ijms19103263

**Published:** 2018-10-20

**Authors:** Xiaoyu Wang, Kaifan Bao, Peng Wu, Xi Yu, Can Wang, Lv Ji, Min Hong

**Affiliations:** Jiangsu Key Laboratory for Pharmacology and Safety Evaluation of Chinese Materia Medica, School of Pharmacy, Nanjing University of Chinese Medicine, Nanjing 210023, China; wangxiaoyu0504@126.com (X.W.); 13255292176@163.com (K.B.); PengWuGo@163.com (P.W.); yuxi1990yaoli@126.com (X.Y.); wangcan1109@126.com (C.W.); ji.lyu.53z@st.kyoto-u.ac.jp (L.J.)

**Keywords:** microarray, microRNA, long non-coding RNA, competing endogenous RNA, atopic dermatitis, recurrence

## Abstract

Atopic dermatitis (AD) is a prevalent inflammatory skin disease characterized by its chronic nature and relapse. Ample evidence suggests that non-coding RNAs play a major role in AD pathogenesis. However, the mechanism remains unknown, particularly in AD recurrence. Dynamic morphological and cytokine changes were measured throughout the whole course of an FITC-induced AD recurrence murine model. Microarray assay and integrative analysis were performed to comprehensively explore long non-coding RNA (lncRNA), messenger RNA (mRNA), and microRNA (miRNA) networks. Our results showed that an AD recurrence model was established. Overall, 5766 lncRNAs, 4025 mRNAs, and 202 miRNAs changed after elicitation, whereas, 419 lncRNAs, 349 mRNAs, and more notably, only 23 miRNAs, were dysregulated in the remission phase. Gene ontology (GO) and KEGG pathway enrichment analyses were used to investigate the potential functions of the dysregulated genes. The altered regulation of seven miRNAs and seven lncRNAs were validated in different stages of the model. The competing endogenous RNA (ceRNA) network inferred that lncRNA humanlincRNA0490+ could compete for miR-155-5p binding, through which it might affect Pkiα expression. Altogether, our findings have provided a novel perspective on the potential roles of non-coding RNAs in AD, and suggest that specific non-coding RNAs could be new therapeutic targets against AD recurrence.

## 1. Introduction

Atopic dermatitis (AD) is a common skin disease with a prevalence of 15–30% in children and 2–10% in adults [1]. AD is defined as a Th2 chronic skin inflammation, which is highly associated with asthma, allergic rhinitis, and immunoglobulin E (IgE)-mediated food reactions [2,3]. A combination of genetic, environmental, and immunological factors are considered to be involved in the pathogenesis of AD [4]. This complex immunological disease often begins in childhood, and can persist, in one form or another, throughout adulthood [5]. Approximately one-third of patients with AD will develop asthma, and two-thirds allergic rhinitis [6]. The persistence and recurrence of AD seriously affects the life quality of patients, which makes it a real threat to health, in this setting. Drugs like corticosteroids, antihistamines, leukotriene modifiers, anticholinergics, β-agonists, and anti-IgE preparations are effective at controlling the symptoms of allergy, however, reducing the relapse of AD remains a worldwide problem, which prompts us to further investigate the pathological mechanism of AD and find new therapeutic targets against it [7,8].

More and more studies suggest that miRNAs act as key regulators in skin inflammation and allergic diseases [9,10]. miRNAs regulate gene expression by inhibiting mRNA translation or initiating the degradation of mRNA targets [11,12]. In contrast to the abundant data linking dysregulation of miRNAs to other allergic diseases, such as asthma, allergic rhinitis, and psoriasis, studies on the role of miRNAs in AD are rather limited [13,14], especially in the process of AD recurrence. In addition, although some lncRNAs have been implicated in the regulation of the immune response [15], their exact function remains unknown. Growing evidence suggests that lncRNAs function as miRNA sponges or ceRNAs, and competitively reduce the binding between miRNAs and their target mRNAs [16]. However, the potential roles of lncRNAs as ceRNAs in AD remains unexplored.

In order to identify the expression profiles of non-coding RNAs in AD recurrence, we first established an AD recurrence murine model and analyzed the differentially expressed lncRNAs, miRNAs, and mRNAs using microarrays. GO and KEGG pathway enrichment, miRNA–mRNA network, and CNC analysis, were performed. Seven lncRNAs and seven miRNAs were confirmed as dysregulated by RT-PCR. In addition, ceRNA expression network construction was performed to discover candidate diagnostic biomarkers and therapeutic targets for AD recurrence.

## 2. Results

### 2.1. Recurrence of AD Appeared Much Quicker and Was More Severe

**Figure 1:**
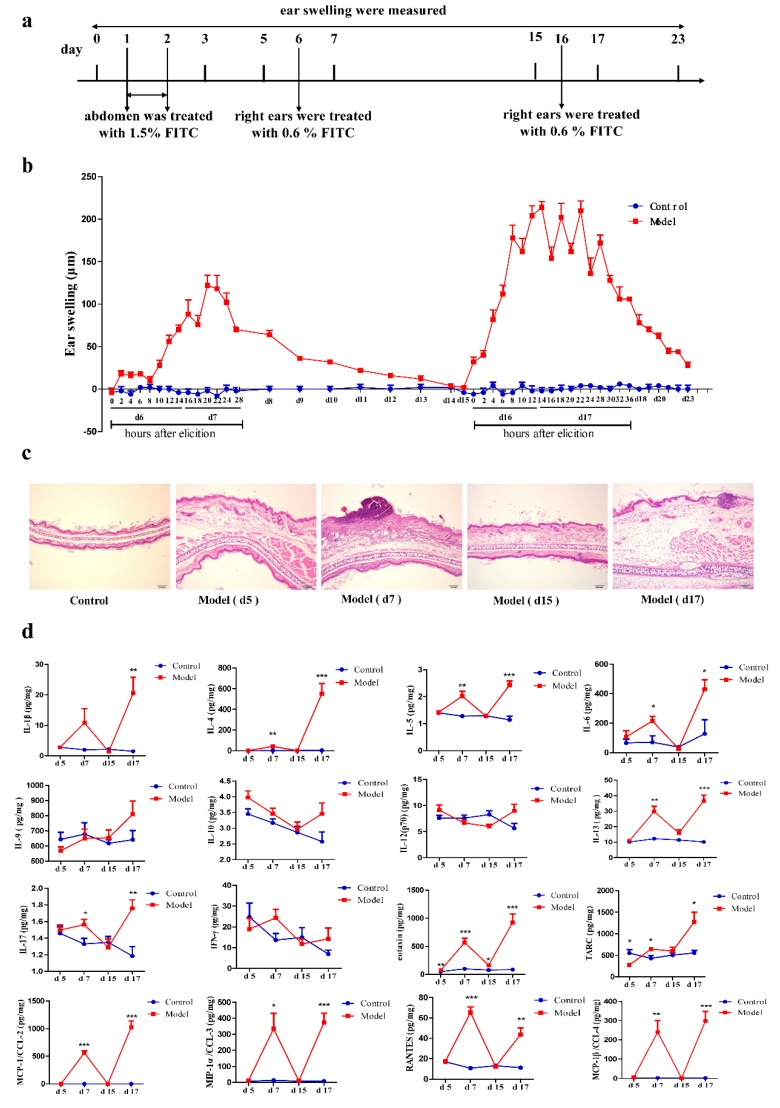
Mouse Th2-mediated atopic dermatitis (AD) recurrence model. (**a**) Flow charts of the FITC-induced AD recurrence model. (**b**) The change of ear thickness is systematically observed during the establishment of the AD recurrence model. The differences between the right and left ear weights were calculated (*n* = 8). (**c**) Histopathological findings with hematoxylin & eosin staining of ear skin sections at different phases of the AD recurrence model (*n* = 5, magnification: 200×). (**d**) The expression of the cytokines IL-1β, IL-4, IL-5, IL-9 IL-10, IL-12(p70), IL-13, IL-17, IFN-γ, TSLP, IL-33, eotaxin, and TARC, as well as the chemokines MCP-1/CCL-2, MIP-1α/CCL-3, MCP-1β/CCL-4, and RANTES, in the ear tissue homogenate of different phases of the AD recurrence model (mean ± SD, *n* = 8, compared with control: * *p* < 0.05, ** *p* < 0.01, *** *p* < 0.001).

To observe the pathological process of AD, we created an FITC-induced AD recurrence murine model using a classical method (Figure 1a). Dynamic morphological change of the ears was measured by testing ear swelling during the whole course of the experiment (Figure 1b). Ear swelling rose to a peak at 20 h after the first elicitation on day 6, and gradually decreased during the following days to the normal level on day 15 (remission). After spontaneous recovery, we elicited a relapse of the inflammatory response by another challenge of FITC on day 16. Ear swelling soared to a higher level compared to the first elicitation, and was steady for a longer period of time, in addition to small fluctuations. Meanwhile, we noticed that ear swelling increased much quicker after the second challenge than the first time, and it exceeded the peak value in just 8 h.

To further investigate the quicker and more severe response in the recurrence phase, we explored the pathology of this model. Histological analysis of ear skin sections demonstrated that the ears did not swell on day 5 after sensitization, but showed severe inflammation on day 7 when exposed locally to FITC. Then, the ears recovered to a steady and non-inflammatory stage on day 15 in the remission phase. By contrast, remarkable ear swelling and inflammatory cell infiltration were observed on day 17 after the second elicitation, which was much more serious than the response observed on day 7 (Figure 1c). In addition, we detected characteristic biomarkers, such as cytokines and chemokines, in ear tissues of different phases with a Luminex bead array assay. Results showed that IL-1β, IL-4, IL-5, IL-6, IL-13, IL-17, eotaxin, MCP-1/CCL-2, MIP-1α/CCL3, MIP-1β/CCL4, RANTES, and TARC, all increased after both elicitations (day 7 and day 17). However, cytokines including IL-2, IL-9, IL-10, IL-12 (p70), and IFN-γ showed no differences between the different phases of AD recurrence. Meanwhile, eotaxin, which induced eosinophil activation, significantly increased in the sensitization phase (Figure 1d). We found most type 2 cytokines and chemokines increased after the first elicitations, and showed a more severe Th2-dominated allergic inflammation after the second elicitation. However, cytokines and chemokines reduced to a normal level in remission (day 15), when inflammation had diminished. This led us to speculate that abnormal expression of RNA, in remission and/or elicitation phase, might be a key factor in the pathogenesis of AD recurrence.

### 2.2. Differentially Expressed miRNAs, lncRNAs, and mRNAs in the Remission and Elicitation Phase of the AD Recurrence Model

**Figure 2:**
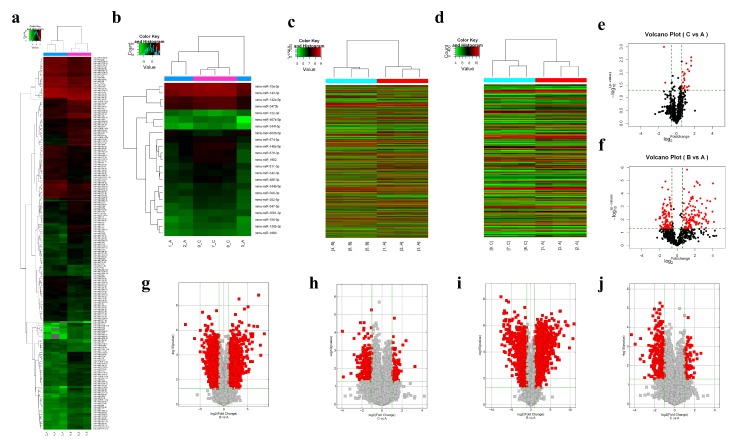
Heatmap and volcano plots showing lncRNA, miRNA, and mRNA expressions in the ear tissue homogenate on the remission and elicitation phase of the AD recurrence model. (**a**,**b**) Hierarchical clustering for differentially expressed miRNAs. (**c**,**d**) Hierarchical clustering for differentially expressed lncRNAs. The color scale shown at the top illustrates the relative expression level of a miRNA and lncRNAs in a specific slide: red, high relative expression; green, low relative expression. (**e**,**f**) Volcano plot of normalized expression levels for differentially expressed miRNAs. (**g**,**h**) Volcano plot of normalized expression levels for differentially expressed lncRNAs. (**i**,**j**) Volcano plot of normalized expression levels for differentially expressed mRNA. The vertical lines correspond to 1.5-fold upregulation and downregulation for miRNAs, 2-fold upregulation and downregulation for miRNAs, and the horizontal line represents a *p* value of 0.05. The red point in the plot represents the differentially expressed miRNAs with statistical significance (A: control; B: elicitation phase; C: remission).

To identify the differentially expressed miRNAs, lncRNAs, and mRNAs in the AD recurrence model, microarray analysis was performed. Flowcharts of mice ear tissue homogenate, collected in the remission and elicitation phases of AD recurrence model, are shown in Figure 1a. We found that 5766 lncRNAs, 4025 mRNAs, and 202 miRNAs changed after elicitation. Meanwhile, 419 lncRNAs, 349 mRNAs, and 23 miRNAs were dysregulated in the remission phase. Compared with the control group (A), miRNAs, lncRNAs and mRNAs significantly changed in the elicitation phase (B) and remission phase (C), with fold change (*FC*) > 1.5 and *p* ≤ 0.05 for miRNA and *FC* > 2.0 and *p* ≤ 0.05 for lncRNA and mRNA. Specifically, 114 upregulated and 88 downregulated miRNAs were identified after elicitation, and 21 miRNAs were upregulated and two downregulated in the remission phase, respectively. In addition, 2394 upregulated and 3372 downregulated lncRNAs were found in the elicitation phase. Meanwhile, 116 and 303 lncRNAs were upregulated and downregulated in the remission phase, respectively. Furthermore, 1829 upregulated and downregulated 2196 mRNAs were identified in the elicitation phase, while 76 mRNAs were upregulated and 273 downregulated in the remission phase. As shown in Figure 2a–d, the heat map represented a two-way hierarchical clustering of differentially expressed miRNAs and lncRNAs. Volcano plot filtering showed the significantly aberrantly expressed miRNAs and lncRNAs, between these two groups (Figure 2e–j). The top 10 upregulated and downregulated miRNA/lncRNA/mRNAs are listed (Tables S1–S3).

### 2.3. Gene Ontology and KEGG Pathway Analysis

**Figure 3:**
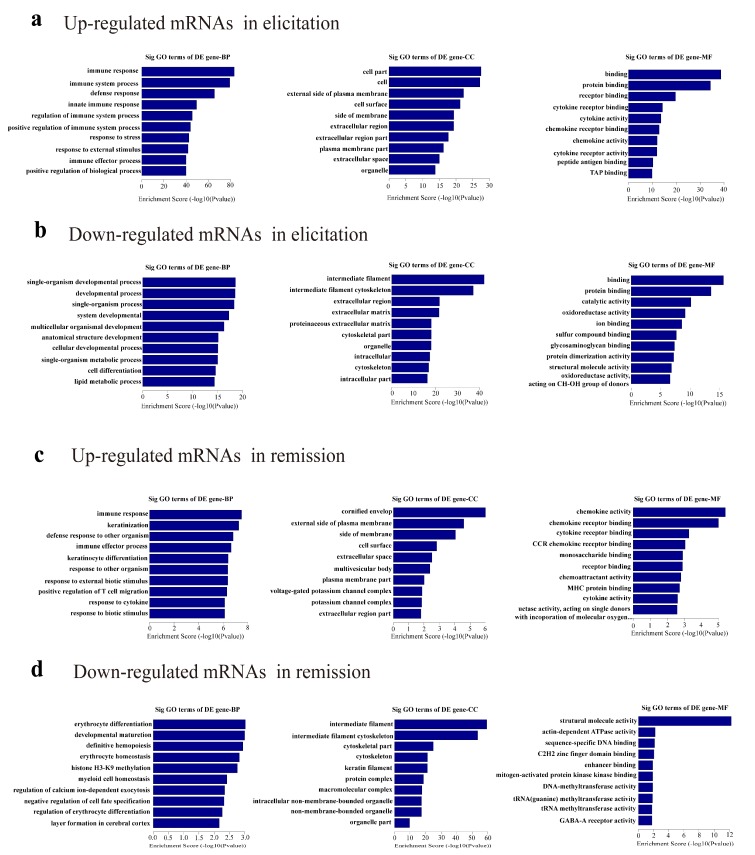
Gene ontology (GO) analysis. (**a**–**d**) GO annotation of upregulated and downregulated mRNAs in elicitation and remission phase of AD recurrence model, with the top ten enrichment scores covering domains of biological processes (BP), cellular components (CC), and molecular functions (MF).

GO enrichment analysis was performed to reveal the roles of significantly dysregulated mRNAs in the elicitation phase and the remission of the AD recurrence model. Our data demonstrated that the upregulated mRNAs of the elicitation phase were genes involved in the immune response, cell parts, and binding (Figure 3a), while the downregulated mRNAs were associated with single-organism developmental processes, intermediate filament, and binding (Figure 3b). Furthermore, the upregulated mRNAs in remission were associated with the immune response, cornified envelope, and chemokine activity (Figure 3c), whereas the downregulated mRNAs in remission were enriched in erythrocyte differentiation, intermediate filament, and structural molecule activity (Figure 3d).

**Figure 4:**
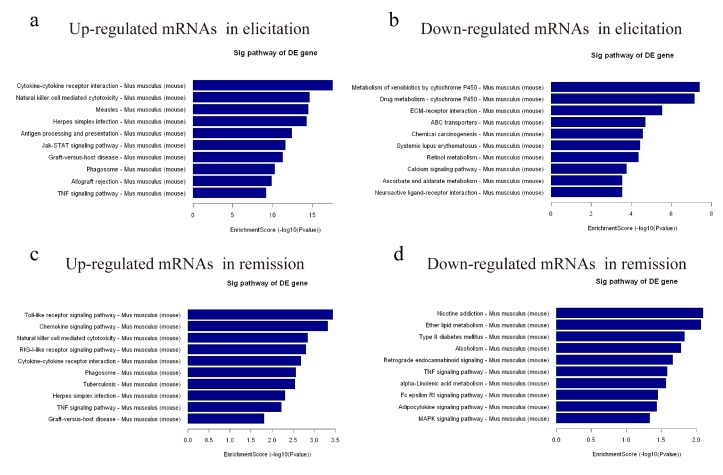
KEGG pathway analysis. (**a**–**d**) KEGG pathway enrichment analysis of upregulated and downregulated mRNAs in the elicitation and remission phase of the AD recurrence model with the top ten enrichment scores.

KEGG pathway enrichment analysis demonstrated the possible involvement of significantly dysregulated mRNAs in related pathways and molecular interactions among genes. Our data showed that the upregulated and downregulated mRNAs were each associated with 10 pathways. The cytokine–cytokine receptor interaction signaling pathway was the most enriched pathway within the set of upregulated mRNAs, and the metabolism of xenobiotics by cytochrome P450 signaling pathway was the most enriched pathway within the downregulated mRNAs in elicitation phase (Figure 4a,b). Furthermore, in remission, the upregulated mRNAs were enriched in the Toll-like receptor signaling pathway, and the downregulated mRNAs were enriched in the nicotine addiction signaling pathway (Figure 4c,d).

### 2.4. miRNA Target Predictions and the miRNA–mRNA Network 

**Figure 5:**
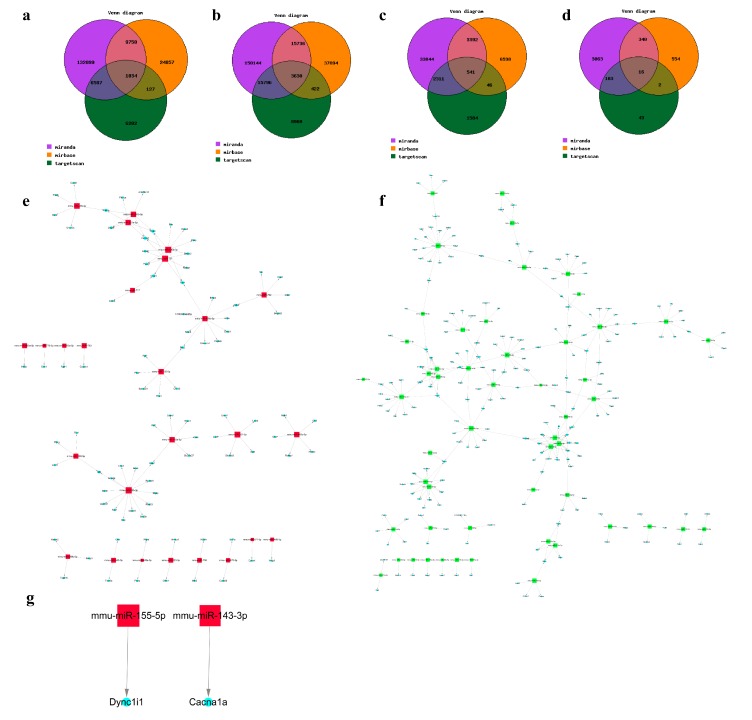
Potential mRNA targets of the differentially regulated miRNAs. (**a**,**b**) Venn diagrams show predicted targets of upregulated and downregulated miRNAs in AD elicitation by three integrated databases (miRanda, miRBase, and TargetScan). (**c**,**d**) Venn diagrams show predicted targets of upregulated and downregulated miRNAs in AD remission by three integrated databases. (**A**: control; **B**: elicitation phase; **C**: remission). (**e**–**g**) Construction of an integrated target gene regulatory network is shown. The network was based on the three integrated databases-derived predicted target genes for upregulated and downregulated miRNAs in elicitation (**e**,**f**) and remission (**g**). (Red represents upregulated miRNAs, green represents downregulated miRNAs, and blue represents mRNAs.)

To predict potential mRNA targets of the differentially regulated miRNAs, two integrated databases (miRanda and TargetScan) were used. Consequently, 6587 target genes were intersected with upregulated miRNAs, and 1034 of them were found in the miRBase database; 15,796 target genes were intersected with downregulated miRNAs, and 1034 of them were found in the miRBase database after elicitation (Figure 5a,b); 2311 target genes were intersected with upregulated miRNAs, and 541 of them were found in the miRBase database; and 103 target genes were intersected with downregulated miRNAs by two databases, and 16 of them were found in the miRBase database (Figure 5c,d).

Based on the miRNA target predictions, and the altered mRNA in elicitation and remission phases, we found that the targets of upregulated miRNAs intersected with downregulated mRNAs in elicitation (Figure 5e). The targets of downregulated miRNAs intersected with upregulated mRNAs (Figure 5f). Meanwhile, mir-155-5p and mir-143-3p were upregulated in remission, and their predicted targets, Dync1i1 and Cacna1a, were downregulated (Figure 5g). However, there was no intersection in remission-downregulated miRNA targets.

### 2.5. CNC Analysis

**Figure 6:**
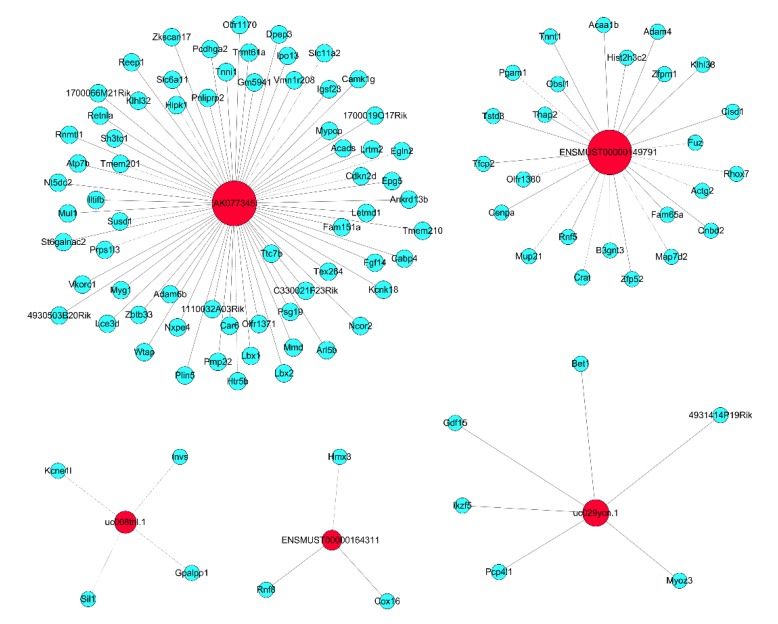
CNC analysis of five lncRNAs with their associated mRNAs. The network is based on the Pearson correlation coefficient (the absolute value of *PCC* ≥ 0.995, and *FDR* < 0.05). The red represent lncRNAs and the light blue represent mRNAs.

Five lncRNAs (AK077345, uc008thl.1, uc029ycn.1, ENSMUST00000164311, and ENSMUST00000149791), which changed markedly, were analyzed by CNC analysis, and as shown in Figure 6, we found potential mRNAs that correlated with these lncRNAs.

### 2.6. Validation of Dysregulated miRNAs in the Remission and Elicitation Phase of the AD Recurrence Model

**Figure 7:**
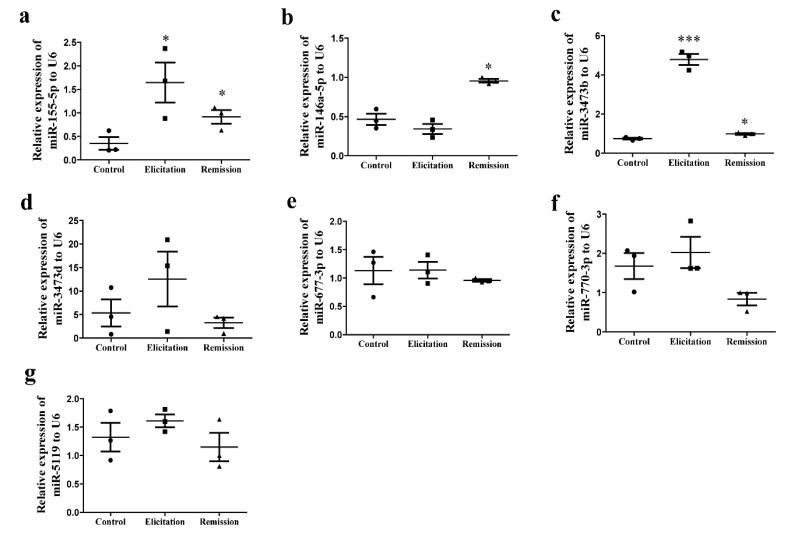
Confirmation of microarray results in the same samples from ear tissue homogenate in the remission and elicitation phase of the AD recurrence model. (**a**–**g**) The expressions of miR-155-5p, miR-146a-5p, miR-3473b, miR-3473d, miR-677-3p, miR-770a-5p, and miR-5119 were detected by qRT-PCR (mean ± SD, *n* = 3, * *p* < 0.05, *** *p* < 0.01 versus A).

To verify our microarray results, seven miRNAs (four upregulated and three downregulated) were selected for further confirmation using qRT-PCR. In accordance with the data from the microarray, miR-155-5p and miR-3473b significantly increased in remission and elicitation phases compared with the control group (Figure 7a,c). In addition, the expression of miR-146a-5p was significantly higher in the remission phase (Figure 7b) while miR-677-3p, miR-770a-5p, and miR-5119 decreased in the remission of AD recurrence (Figure 7e–g).

### 2.7. Validation of Dysregulated lncRNAs in the Remission of AD Recurrence Model

**Figure 8:**
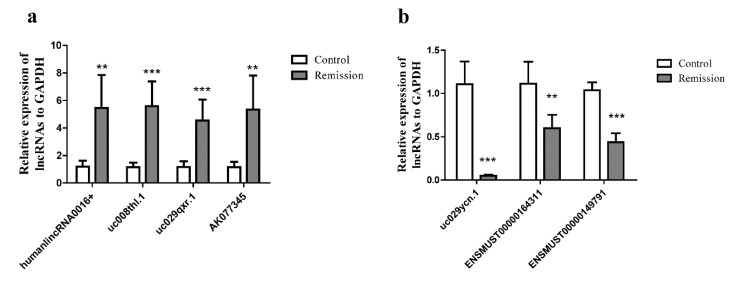
Confirmation of microarray results in the same samples from the ear tissue homogenate in the remission phase of the AD recurrence model. (**a**,**b**) lncRNAs humanlincRNA0016+, uc008thl.1, uc029qxr.1, AK077345, uc029ycn.1, ENSMUST00000164311, and ENSMUST00000149791 were detected by qRT-PCR (mean ± SD, *n* = 3, ** *p* < 0.01, *** *p* < 0.001 versus A).

Based on bioinformatic prediction results, we selected seven lncRNAs identified from the ear tissue of mice to validate lncRNAs via qRT-PCR analysis. LncRNAs humanlincRNA0016+, uc008thl.1, uc029qxr.1, and AK077345 were upregulated (Figure 8a), while uc029ycn.1, ENSMUST00000164311, and ENSMUST00000149791 were downregulated (Figure 8b).

### 2.8. Construction of a ceRNA Network

**Figure 9:**
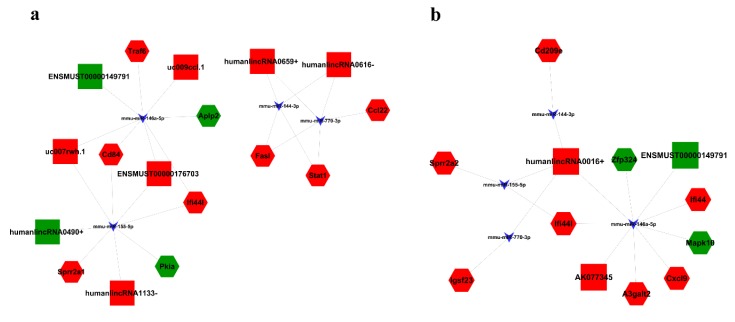
Competing endogenous RNA network in AD mice. miRNA-mediated lncRNA–mRNA ceRNA network. The competing endogenous RNA network is based on lncRNA/miRNA, lncRNA/mRNA, and miRNA/mRNA interactions. (**a**) The competing endogenous RNA network in the elicitation phase of AD. (**b**) The competing endogenous RNA network in remission of AD. (The triangles represent miRNAs, squares represent lncRNAs, and hexagons represent mRNAs. The red indicates an upregulation in the array data; the green indicates a downregulation in the array data.)

Since lncRNAs can interact with miRNAs through their response elements within a ceRNA network, we constructed a ceRNA network based on co-expressed miRNAs–mRNAs, miRNAs–lncRNAs, and lncRNAs–mRNAs. In the elicitation phase, lncRNA humanlincRNA0490+ competed for binding to miR-155-5p, thereby affecting Pkiα expression (Figure 9a). In the remission phase, lncRNA ENSMUST00000149791 competitively bound with miR-146a-5p, which led to an increase of MAPK10, and lncRNA AK077345 could increase Cxcl9 by binding competitively with miRNA-146a-5p. HumanlincRNA0016+ acted as a ceRNA and competitively bound with miR-155-5p or miRNA-146a-5p, which contributed to elevated Ifi44l expression (Figure 9b).

## 3. Discussion

AD is a refractory cutaneous disorder associated with a defective skin barrier and a mixed Th1/Th2 inflammatory response, resulting in susceptibility to cutaneous infections and prominent pruritis [17]. Despite extensive research, there is no effective method to prevent the occurrence and relapse of AD, because the existing therapies for AD aim to control the symptoms, rather than cure the disease [18]. Therefore, further characterization of the molecular mechanisms contributing to the pathogenesis of AD is of great importance and could, ultimately, help in the development of more personalized therapeutic approaches against this disease.

In recent studies, ncRNAs have been found to have critical roles in regulating key pathogenic mechanisms in allergic inflammation. However, the functions of miRNAs and lncRNAs in AD have been described only in a small number of studies. Previous studies have shown that miR-21, miR-146, and miR-223 are not only increased in several other allergic disorders, but also upregulated in AD [19]. Moreover, miR-155 has been identified as overexpressed in skin from AD patients compared to healthy controls, and by downregulating cytotoxic T lymphocyte-associated antigen (CTLA)-4, a negative regulator of T-cell function, miR-155 could influence the development of AD [20,21]. In addition, studies have shown that expression levels of miRNA-21 are significantly downregulated in AD and psoriasis [22]. Differentiation and activation of immune cells, including T cells, B cells, macrophages, and NK cells, have been considered in correlation with lncRNAs, which are also positioned to play an essential role in autoimmune diseases, such as rheumatoid arthritis and systemic lupus erythematosus [15]. Therefore, increasing evidence raises the possibility of elucidating how the roles of miRNAs and lncRNAs in allergic disease contribute to understanding the pathogenesis of AD recurrence.

In this study, an FITC-induced AD recurrence murine model was established for observing the occurrence and development of AD. The dynamic changes of ear swelling were measured during the whole course of the experiment. We found that epidermis swelling and infiltration of inflammatory cells was serious after the first elicitation, then gradually recovered to a normal level (remission) and, finally, soared to much more severe swelling after the second elicitation. Luminex bead array assay showed that most type 2 cytokines (IL-4, IL-5, and IL-13) increased after the first elicitation, and showed an augmented Th2-dominated allergic inflammation after the second elicitation. However, cytokines and chemokines recovered to normal in remission (day 15) with negligible inflammation. These results showed that we successfully established an AD recurrence model to investigate the abnormal expression of genes in remission and elicitation phases, especially those genes which could be the key to exploring the pathogenesis of AD recurrence.

We also performed lncRNA, miRNA, and mRNA expression profiling in the remission and elicitation phase of AD recurrence. For the first time, our study has reported the expression of lncRNAs, miRNAs, and mRNAs in an AD recurrence murine model. From the microarray expression profiles, 5766 lncRNAs and 4025 mRNAs, as well as 202 miRNAs, were identified as aberrantly expressed in the elicitation phase, whereas 419 lncRNAs, 349 mRNAs, and only 23 miRNAs were dysregulated in remission. These results indicated that even when the allergic inflammation had been determined as negligible, there were still many abnormally expressed RNAs in the remission phase, which might be a pivotal factor in the pathogenesis of AD recurrence.

Based on GO enrichment analysis results, differentially expressed mRNAs were mainly associated with the immune response in biological processes, and enriched in chemokine activity and associated with structural molecule activity in the molecular function. KEGG pathway analysis for the differentially expressed mRNAs revealed 10 pathways that could be implicated in AD, including Toll-like receptor, calcium, TNF, MAPK, and PI3K signaling pathways. These pathways are known to be involved in the occurrence and development of the inflammatory response, which suggests that they might be involved in the pathogenesis of AD [23,24].

Furthermore, putative target genes of miRNAs were inferred by three databases, and combined with the constructed miRNA-mRNA network; these target genes were also differentially expressed in an mRNA array. To illustrate, Dync1i1 is the predicted target gene of mir-155-5p, which was also found to be downregulated in the remission phase. These data revealed key RNAs in AD recurrence. In addition, a CNC co-expression network was constructed to predict the function of lncRNAs, and we found that many dysregulated lncRNA significantly correlated with the expression of dozens of protein-coding mRNAs. In consequence, these lncRNAs might be correlated with AD by regulating co-expression genes.

To verify our microarray results, seven miRNAs (miR-155-5p, miR-146a-5p, miR-3473b, miR-3473d, miR-677-3p, miR-770a-5p, and miR-5119) and seven dysregulated lncRNAs (humanlincRNA0016+, uc008thl.1, uc029qxr.1, AK077345, uc029ycn.1, ENSMUST00000164311, and ENSMUST00000149791) were selected for further confirmation. Results showed that miR-146a, miR-155-5p, and miR-3473b significantly increased, while miR-770a-5p and miR-5119 were substantially downregulated in remission. LncRNAs humanlincRNA0016+, uc008thl.1, uc029qxr.1, and AK077345 were upregulated, while uc029ycn.1, ENSMUST00000164311, and ENSMUST00000149791 were downregulated. The results were consistent with those of the microarray analyses, confirming the reliability of the microarray results.

Studies have shown that ceRNAs have an important influence on regulating gene expression at the post-transcriptional level, and are involved in many diseases. In this study, we also constructed a ceRNA network based on miRNA-mRNA, miRNA-lncRNA, and lncRNA-mRNA co-expression patterns. Notably, the ceRNA, lncRNA humanlincRNA0490+, competed for binding to miR-155-5p, which subsequently affected Pkiα expression. Our results showed that miR-155-5p increased, and Pkiα decreased, in an AD model. Considering our prediction that Pkiα, with the highest score, was the target of miR-155-5p, it would be worthy to perform further validation and functional examination experiments to reveal the underlying mechanisms of humanlincRNA0490+ in AD.

The current therapies for allergic diseases are still inefficient in controlling severe AD or asthma, and more effective therapies are urgently required [18]. One direction in the development of novel therapeutics for allergic diseases is the biological modification of immune responses for better and more individualized control [25]. Indeed, ncRNAs are considered to have great potential as novel target molecules for the development of biological treatment modalities. Since the functions of ncRNAs are shaped by evolution, miRNA or lncRNA-based therapeutics would have a low toxicity, and few side effects [26].

In summary, we pioneered an AD recurrence model and revealed that the RNA (miRNAs, lncRNAs, and mRNAs) expression profile greatly contributes to the pathogenesis mechanism of AD. Our data indicate that aberrantly expressed mRNAs, miRNAs, or lncRNAs may serve as biomarkers for diagnosing different stages of AD, especially in the remission phase, and might be novel therapeutic targets against AD recurrence.

## 4. Materials and Methods

### 4.1. Animals

BALB/c mice were purchased from Shanghai SLAC Laboratory Animal Co., Ltd. (Shanghai, China). Mice at 6–8 weeks of age were raised at Nanjing University of Chinese Medicine under specific pathogen-free conditions at 18–25 °C and 50–60% humidity. All procedures involving animals were approved by the Animal Care and Use Committee of Nanjing University of Chinese Medicine (ACU-41, 29-12-2014) and were performed strictly according to the Guide for the Care and Use of Laboratory Animals.

### 4.2. Mouse Th2-Mediated AD Recurrence Model

After acclimatization for 3 days, the abdomens of mice were shaved by an area of about 3 × 3 cm^2^. The abdominal skin of the mice was sensitized with 1.5% FITC (Sigma-Aldrich, St. Louis, MO, USA) in 80 µL of acetone and dibutyl phthalate (1:1; vehicle) on days 1 and 2, and the right ear was treated with 20 µL of 0.6% FITC solution on days 6 and 16. Ear swelling was measured daily with a thickness gauge (7301; Mitutoyo, Kawasaki, Japan), from day 0 to day 23 except days 6, 7, 16, and 17. After the first elicitation on day 6, ear swelling was measured every 2 h for 28 h in total, until the ear swelling showed a declining trend and eight mice were sacrificed to collect the samples for the elicitation phase on day 7. Similarly, ear swelling was measured every 2 h for 36 h altogether, until the measurement presented a tenuous decreasing trend, and eight mice were sacrificed for recurrence phase on day 17. On days 5 and 15, mice were sacrificed to collect samples for sensitization and remission phases. The histopathological changes in the ears were examined with hematoxylin and eosin (H & E) staining.

### 4.3. Cytokine and Chemokine Analysis in Ear Homogenates

Both ears of each mouse were removed and ground to homogenates in ice-cold phosphate-buffered saline (PBS), which contained 1× protease inhibitor (Merck Millipore, Billerica, MA, USA), and the homogenates were centrifuged at 1000× *g* for 10 min at 4 °C. The supernatants were stored at −80 °C until analysis. The ear tissue supernatants were assayed for IL-1β, IL-4, IL-5, IL-9 IL-10, IL-12(p70), IL-13, IL-17, IFN-γ, TSLP, IL-33, eotaxin, and TARC, as well as the chemokines MCP-1/CCL-2, MIP-1α/CCL-3, MCP-1β/CCL-4, and RANTES, using Mouse Cytokine/Chemokine Magnetic Bead Panel II kit (Millipore, Billerica, MA, USA), according to manufacturer’s instructions. The plates were read using a Luminex^®^200^TM^ Total System machine (Luminex Corp, Austin, TX, USA). Data were analyzed using the data ProcartaPlex Analyst 1.0.

### 4.4. RNA Extraction

Total RNA from the ear tissues of each group were homogenized in TRIzol (Invitrogen, Carlsbad, CA, USA) and purified with an RNeasy Mini Kit (Qiagen, Duesseldorf, Germany), according to manufacturer’s instructions. RNA quality and quantity were measured by using a Nanodrop spectrophotometer (ND-1000, Nanodrop Technologies, Waltham, MA, USA), and RNA Integrity was determined by gel electrophoresis.

### 4.5. LncRNA and mRNA Microarray

Arraystar Mouse LncRNA Microarray V3.0 is designed for the global profiling of mouse lncRNAs and protein-coding transcripts. About 35,923 lncRNAs and 24,881 coding transcripts can be detected by our third-generation lncRNA microarray. The microarray hybridization, collection of expression data, and analysis of microarray data were performed by KangCheng Bio-Tech (Shanghai, China).

### 4.6. RNA Labeling and Array Hybridization

Sample labeling and array hybridization were performed using the Agilent One-Color Microarray-Based Gene Expression Analysis protocol (Agilent Technology, Santa Clara, CA, USA). Briefly, mRNA was purified from total RNA after removal of rRNA (mRNA-ONLY™ Eukaryotic mRNA Isolation Kit, Epicentre, Madison, WI, USA). Then, each sample was amplified and transcribed into fluorescent cRNA along the entire length of the transcripts without 3′ bias, utilizing a random priming method (Arraystar Flash RNA Labeling Kit, Arraystar, Rockville, MD, USA). The labeled cRNAs were purified by an RNeasy Mini Kit (Qiagen). The concentration and specific activity of the labeled cRNAs (pmol Cy3/µg cRNA) were measured using a NanoDrop ND-1000. One microgram of each labeled cRNA was fragmented by adding 5 µL 10× blocking agent and 1 μL of 25× fragmentation buffer, then, the mixture was heated for 30 min at 60 °C. Finally, 25 µL 2× GE hybridization buffer was added to dilute the labeled cRNA, and 50 µL of hybridization solution was dispensed into the gasket slide and assembled to the lncRNA expression microarray slide. The slides were incubated for 17 h at 65 °C in an Agilent Hybridization Oven. The hybridized arrays were washed, fixed, and scanned using the Agilent DNA Microarray Scanner (part number G2505C).

### 4.7. miRNA Labeling and Array Hybridization

RNA labeling and array hybridization were performed according to the Exiqon manual. The miRCURY™ Hy3™/Hy5™ Power Labeling Kit (Exiqon, Vedbaek, Denmark) was used for miRNA labeling after quality control. RNA (1 µg in 2 µL of water) was combined with 1 µL of calf intestine phosphatase (CIP) buffer and CIP enzyme (Exiqon), and incubated for 30 min at 37 °C. The reaction was terminated by incubation for 5 min at 95 °C. Then, 3 µL of labeling buffer, 1.5 µL of fluorescent label (Hy3TM), 2 µL of DMSO, and 2 µL of labeling enzyme were added to the mixture. The labeling reaction was incubated for 1 h at 16 °C, and then terminated by incubation for 15 min at 65 °C. After stopping the labeling procedure, the Hy3™-labeled samples were hybridized on the miRCURYTM LNA Array (v.18.0) (Exiqon) according to the array manual. The Hy3™-labeled samples (25 µL), mixed with 25 µL hybridization buffer, were first denatured for 2 min at 95 °C, incubated on ice for 2 min, and then hybridized to the microarray for 20 h at 56 °C in a 12-Bay Hybridization System (Hybridization System-Nimblegen Systems, Inc., Madison, WI, USA). Following hybridization, the slides were washed several times with a wash buffer kit (Exiqon) and then scanned using an Axon GenePix 4000B microarray scanner (Axon Instruments, Foster City, CA, USA).

### 4.8. Array Analysis and Normalization

For RNAs, Agilent Feature Extraction software (version 11.0.1.1) was used to analyze acquired array images. Quantile normalization and subsequent data processing were performed using the GeneSpring GX v12.1 software package (Agilent Technologies, Santa Clara, CA, USA). After quantile normalization of the raw data, lncRNAs and mRNAs of at least three out of the 12 samples, with flags in Present or Marginal (“All Target Values”), were chosen for further data analysis. Differentially expressed lncRNAs and mRNAs with statistical significance between the two groups were identified through *p* value/FDR filtering. Differentially expressed lncRNAs and mRNAs between the two samples were identified through FC filtering. Hierarchical clustering and combined analysis were performed using homemade scripts. For miRNAs, scanned images were imported into GenePix Pro 6.0 software (Axon) for grid alignment and data extraction. Replicated miRNAs were averaged, and miRNAs with intensities ≥30 in all samples were chosen for calculating the normalization factor. Expressed data were normalized using the median normalization. After normalization, significant differentially expressed miRNAs between the two groups were identified through FC and *p* values. Differentially expressed miRNAs between two samples were filtered through FC. Finally, hierarchical clustering was performed to show distinguishable miRNA expression profiling among samples.

### 4.9. Gene Ontology Analysis and KEGG Pathway Analysis

We conducted gene ontology (GO) analysis to provide annotation of gene and gene product attributes in any organism (http://www.geneontology.org). The ontology covers three domains: biological processes, cellular components, and molecular functions. The *p* value (≤0.05) denotes the significance of GO term enrichment among differentially expressed genes. We also performed KEGG pathway analysis to harvest pathway clusters covering the molecular interaction and reaction networks in differentially regulated gene profiling. The *p* value (≤0.05) denotes the significance of the pathway correlations. The GO and KEGG pathways analyses were performed by KangCheng Bio-Tech, Shanghai, China.

### 4.10. miRNA Target Prediction

miRNA binding sites were predicted using homemade software based on TargetScan 6.0 and miRanda v3.3a. The miRNA binding sites predicted by the two software were combined, and the energy score and pair score of sites predicted by TargetScan were also calculated by miRanda.

### 4.11. Quantitative Real-Time PCR

Ear tissues of each group were homogenized in 1 mL of TRIzol (Invitrogen). The total RNA was isolated according to the manufacturer’s protocol. SYBR green PCR Master Mix (Arraystar) was used for real-time PCR analysis. All reactions were run on a ViiA 7 Real-time PCR System (Applied Biosystems, Carlsbad, CA, USA). The primers used in this study are summarized in Table 1. All lncRNA expression data were normalized to GAPDH, and miRNAs were normalized to U6. The relative expression of the genes was calculated using the 2^−ΔΔ*C*t^ method. 

### 4.12. CNC Analysis

The CNC analysis was based on the Pearson correlation coefficient (*PCC*) between the expression levels of coding and non-coding genes. We subsequently based screening on the Pearson correlation coefficient using the selection parameters *PCC* ≥ 0.995 and *FDR* < 0.05. Cytoscape (v3.4.0) was used to illustrate the co-expression network. Analyses were performed by KangCheng Bio-Tech, Shanghai, China.

### 4.13. Construction of the ceRNA Network

We constructed the ceRNA network based on two criteria: LncRNAs that were dysregulated by an *FC* of ≥2.0 and *p* value <0.05 that significantly correlated with the miRNAs predicted target genes; and lncRNAs that possessed miRNAs MREs, as predicted by RNA22 (https://cm.jefferson.edu/rna22/Precomputed/) and PITA (http:// genie.weizmann.ac.il/pubs/mir07/mir07_data.html).

### 4.14. Statistical Analysis

Data were expressed as means ± SD. One-way ANOVA analysis was used for multiple group comparisons, and Dunnett’s test was used for comparison between two groups, with GraphPad Prism 5 (GraphPad Software, San Diego, CA, USA). Statistical significance was set at *p* < 0.05.

## Figures and Tables

**Table 1:** PCR primers used in this study.

Name	Sequence
U6	F: 5′-GCTTCGGCAGCACATATACTAAAAT-3′ R: 5′-CGCTTCACGAATTTGCGTGTCAT-3′
mmu-miR-3473d	GSP: 5′-GCCACTGAGCCACTTTCCA-3′ R: 5′-GTGCGTGTCGTGGAGTCG-3′
mmu-miR-155-5p	GSP: 5′-GGGGGTAATGCTAATTGTGAT-3′ R: 5′-CAGTGCGTGTCGTGGAGT-3′
mmu-miR-3473b	GSP: 5′-GGGGAAGGCTGGAGAGATG-3′ R: 5′-GTGCGTGTCGTGGAGTCG-3′
mmu-miR-677-3p	GSP: 5′-AAGCCAGATGCCGTTCCT-3′ R: 5′-GTGCGTGTCGTGGAGTCG-3′
mmu-miR-770-5p	GSP: 5′-GGGAAGCACCACGTGTCTG-3′ R: 5′-GTGCGTGTCGTGGAGTCG-3′
mmu-miR-5119	GSP: 5′-GGGGGTCATCTCATCCTGG-3′ R: 5′-GTGCGTGTCGTGGAGTCG-3′
mmu-miR-146a-5p	GSP: 5′-GGGTGAGAACTGAATTCC-3′ R: 5′-TGCGTGTCGTGGAGTC-3′
humanlincRNA0016+	F: 5′-CCACACCCATGCTCTATGTG-3′ R: 5′-GACAAAGCAACAGCGAAACA-3′
uc008thl.1	F: 5′-ATGCTCAATTCTCGCCAAGT-3′ R: 5′-CAGTTTCAGGGCCACACATA-3′
uc029qxr.1	F: 5′-AAGCTGATGACTAAGATCCCT-3′ R: 5′-GGCTCATCTTGGACCACTCTC-3′
AK077345	F: 5′-ACCAAGCTGCCAATCCATAG-3′ R: 5′-TTGGGTGGCTGTTTTCTACC-3′
uc029ycn.1	F: 5′-AGTTGTGAGGGAGAGGGACA-3′ R: 5′-CGAAAGAGCCTCCTCTGAAA-3′
ENSMUST00000164311	F: 5′-TTCATCAGCAGCACATAGGC-3′ R: 5′-TGTTTCCCCGTAACACCAAT 3′
ENSMUST00000149791	F: 5′-GTTGCCAACCTGGAGAAAGA-3′ R: 5′-TCCAGGTTTTGCTTGGTTCT-3′
GAPDH	F: 5′-GGTTGTCTCCTGCGACTTCA-3′ R: 5′-TGGTCCAGGGTTTCTTACTCC-3′

## Supplementary Materials

Supplementary materials can be found at http://www.mdpi.com/1422-0067/19/10/3263/s1.
