# Supplementary material for: Integrative Analysis of lncRNAs, miRNAs, and mRNA-Associated ceRNA Network in an Atopic Dermatitis Recurrence Model
Source: Int J Mol Sci. 2018 Oct 20;19(10):3263. doi: 10.3390/ijms19103263 (PMC6214145; doi:10.3390/ijms19103263)
Supplement: Supplementary file 1 [file ijms-19-03263-s001.zip › ijms-362907-sup.pdf]

Supplementary Material

Integrative Analysis of lncRNAs, miRNAs, and mRNA-Associated ceRNA Network in an Atopic Dermatitis Recurrence Model

Table S1. The top 10 upregulated and downregulated mRNAs in the elicitation and remission phases.

mRNAs in Elicitation			mRNAs in Remission		
GeneSymbol	Fold Change	Regulation	GeneSymbol	Fold Change	Regulation
Il19	2020.955	up	Sprr2a2	6.190	up
Gm4841	1730.448	up	Klrc1	4.717	up
Saa3	917.033	up	Ubash3a	4.670	up
Cxcl9	710.958	up	Saa3	3.939	up
Sprr2a2	561.445	up	Gm12250	3.856	up
Tdgf1	441.431	up	Gimap3	3.674	up
Gm12250	345.388	up	Klk1b16	3.646	up
Fgf23	208.492	up	Klrc1	3.631	up
Gsdmc	182.459	up	Cxcl10	3.316	up
Sell	172.297	up	Olfr825	3.213	up
Gm11568	202.135	down	Olfr678	19.776	down
Gm11559	196.005	down	Krtap7-1	15.323	down
Lyg2	167.089	down	Krtap19-4	10.694	down
Krtap21-1	123.587	down	Herc2	10.523	down
Krtap9-3	121.480	down	Krtap19-1	9.242	down
Adra2c	119.387	down	Ccdc175	8.347	down
Gm11570	118.620	down	Vmn2r88	7.922	down
Krtap3-1	113.231	down	Krtap22-2	5.998	down
2310061N02Rik	110.424	down	Olfr1215	5.910	down
Krtap2-4	109.083	down	Krtap19-3	5.869	down

Table S2. The top 10 upregulated and downregulated miRNAs in the elicitation and remission phases.

miRNAs in Elicitation			miRNAs in Remission		
Name	Fold Change	Regulation	Name	Fold Change	Regulation
mmu-miR-5127	19.644	up	mmu-miR-146a-5p	3.112	up
mmu-miR-465-3p	17.664	up	mmu-miR-155-5p	3.090	up
mmu-miR-3473b	16.304	up	mmu-miR-1902	3.082	up
mmu-miR-3474	15.643	up	mmu-miR-344f-5p	2.667	up
mmu-miR-714	13.159	up	mmu-miR-878-3p	2.595	up
mmu-miR-5128	11.535	up	mmu-miR-547-5p	2.376	up
mmu-miR-339-3p	10.875	up	mmu-miR-467b-5p	2.357	up
mmu-miR-1934-5p	10.530	up	mmu-miR-489-3p	2.239	up
mmu-miR-292a-5p	9.814	up	mmu-miR-511-3p	2.136	up
mmu-miR-139-5p	9.588	up	mmu-miR-382-5p	1.903	up
mmu-miR-99a-3p	0.189	down	mmu-miR-122-3p	0.377	down
mmu-miR-33-5p	0.248	down	mmu-miR-883b-5p	0.416	down
mmu-miR-872-5p	0.260	down			

mmu-miR-144-3p	0.266	down
mmu-miR-31-5p	0.268	down
mmu-miR-218-5p	0.287	down
mmu-miR-148a-3p	0.310	down
mmu-miR-26a-2-3p	0.314	down
mmu-miR-214-5p	0.315	down
mmu-miR-28a-5p	0.319	down

Table S3. The top 10 upregulated and downregulated LncRNAs in the elicitation and remission phases.

**LncRNAs in Elicitation**			**LncRNAs in Remission**		
**GeneSymbol**	**Fold Change**	**Regulation**	**GeneSymbol**	**Fold Change**	**Regulation**
Gm11597	367.747	up	humanlincRNA0016	9.731	up
BC023105	222.588	up	Themis	5.286	up
Mx1	208.465	up	TCR-alpha chain	4.246	up
Fos	160.154	up	Gm20500	3.761	up
BC023105	137.121	up	AK042944	3.443	up
Oasl2	79.501	up	TCR-alpha chain	3.412	up
Gbp2	68.574	up	humanlincRNA0728	3.240	up
Gm5970	67.719	up	humanlincRNA2331	3.202	up
Cd300lf	64.662	up	XLOC_018617	3.187	up
Cd300lf	64.662	up	XLOC_026162	3.122	up
AK078248	2.000	down	uc.65	15.499	down
humanlincRNA0488	2.001	down	XLOC_021493	14.527	down
DQ699600	2.001	down	AK139454	8.533	down
uc.7	2.002	down	AK046482	6.907	down
uc.7	2.002	down	Fam160b1	6.891	down
Ppm1m	2.002	down	AK040222	5.894	down
Ppm1m	2.002	down	humanlincRNA2350	5.571	down
AK077696	2.002	down	AK141077	5.466	down
AK041883	2.003	down	Accn1/Mpped2	5.442	down
Gm13131	2.003	down	XLOC_015762	5.421	down
